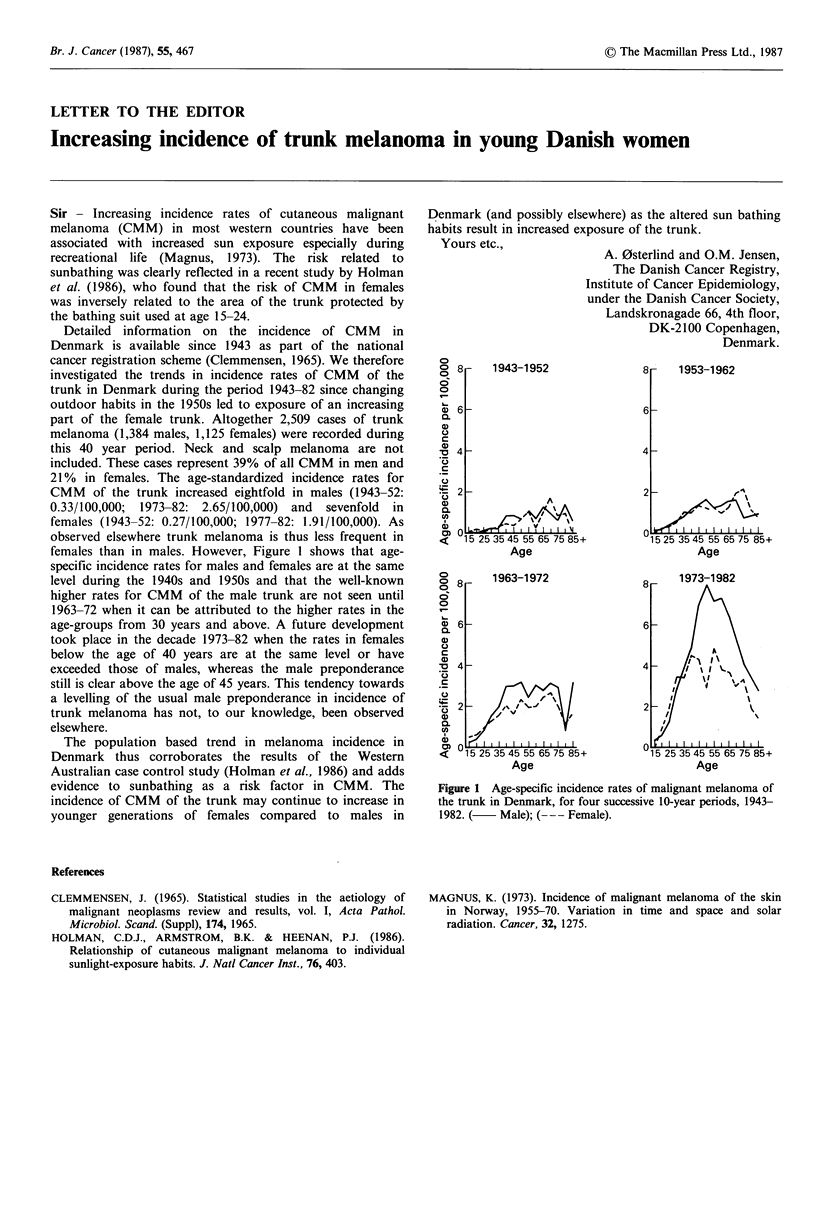# Increasing incidence of trunk melanoma in young Danish women.

**DOI:** 10.1038/bjc.1987.92

**Published:** 1987-04

**Authors:** A. Osterlind, O. M. Jensen


Br. J. Cancer (1987), 55, 467 © The Macmillan Press Ltd., 1987

LETTER TO THE EDITOR

### Increasing incidence of trunk melanoma in young Danish women

**Sir** – Increasing incidence rates of cutaneous malignant melanoma (CMM) in most western countries have been associated with increased sun exposure especially during recreational life (Magnus, 1973). The risk related to sunbathing was clearly reflected in a recent study by Holman *et al.* (1986), who found that the risk of CMM in females was inversely related to the area of the trunk protected by the bathing suit used at age 15–24.

Detailed information on the incidence of CMM in Denmark is available since 1943 as part of the national cancer registration scheme (Clemmensen, 1965). We therefore investigated the trends in incidence rates of CMM of the trunk in Denmark during the period 1943–82 since changing outdoor habits in the 1950s led to exposure of an increasing part of the female trunk. Altogether 2,509 cases of trunk melanoma (1,384 males, 1,125 females) were recorded during this 40 year period. Neck and scalp melanoma are not included. These cases represent 39% of all CMM in men and 21% in females. The age-standardized incidence rates for CMM of the trunk increased eightfold in males (1943–52: 0.33/100,000; 1973–82: 2.65/100,000) and sevenfold in females (1943–52: 0.27/100,000; 1977–82: 1.91/100,000). As observed elsewhere trunk melanoma is thus less frequent in females than in males. However, Figure 1 shows that age-specific incidence rates for males and females are at the same level during the 1940s and 1950s and that the well-known higher rates for CMM of the male trunk are not seen until 1963–72 when it can be attributed to the higher rates in the age-groups from 30 years and above. A future development took place in the decade 1973–82 when the rates in females below the age of 40 years are at the same level or have exceeded those of males, whereas the male preponderance still is clear above the age of 45 years. This tendency towards a levelling of the usual male preponderance in incidence of trunk melanoma has not, to our knowledge, been observed elsewhere.

The population based trend in melanoma incidence in Denmark thus corroborates the results of the Western Australian case control study (Holman *et al.*, 1986) and adds evidence to sunbathing as a risk factor in CMM. The incidence of CMM of the trunk may continue to increase in younger generations of females compared to males in Denmark (and possibly elsewhere) as the altered sun bathing habits result in increased exposure of the trunk.

Yours etc.,

A. Østerlind and O.M. Jensen,
The Danish Cancer Registry,
Institute of Cancer Epidemiology,
under the Danish Cancer Society,
Landskronagade 66, 4th floor,
DK-2100 Copenhagen,
Denmark.

**Figure 1** Age-specific incidence rates of malignant melanoma of the trunk in Denmark, for four successive 10-year periods, 1943–1982. (—— Male); (– – – Female).

**References**

CLEMMENSEN, J. (1965). Statistical studies in the aetiology of malignant neoplasms review and results, vol. I, *Acta Pathol. Microbiol. Scand.* (Suppl), **174**, 1965.

HOLMAN, C.D.J., ARMSTROM, B.K. & HEENAN, P.J. (1986). Relationship of cutaneous malignant melanoma to individual sunlight-exposure habits. *J. Natl Cancer Inst.*, **76**, 403.

MAGNUS, K. (1973). Incidence of malignant melanoma of the skin in Norway, 1955–70. Variation in time and space and solar radiation. *Cancer*, **32**, 1275.